# CNS tumor with *CREBBP::BCORL1* Fusion and pathogenic mutations in *BCOR* and *CREBBP*: expanding the spectrum of *BCOR*-altered tumors

**DOI:** 10.1186/s40478-024-01726-x

**Published:** 2024-01-12

**Authors:** Valeria Barresi, Antonello Cardoni, Evelina Miele, Lucia Pedace, Barbara Masotto, Claudia Nardini, Sabina Barresi, Sabrina Rossi

**Affiliations:** 1 Department of Diagnostics and Public Health, University of Verona, Verona, Italy; 2 Pathology Unit, Bambino Gesù Children’s Hospital, IRCCS, Rome, Italy; 3 Oncohematology Research Area, Genetics and Epigenetics of tumors, IRCCS Bambino Gesù Children’s Hospital, Rome, Italy; 4 Unit of Cranial Posterior Fossa Surgery, University and Hospital Trust of Verona, Verona, Italy; 5 Department of Diagnostics and Public Health, Policlinico G.B. Rossi, P.le L.A. Scuro, 10, Verona, 37138 Italy

**Keywords:** CNS tumor, *BCOR*, *BCORL1*, *CREBBP*, Fusion, Ependymoma

## Abstract

The fifth edition of the World Health Organization (WHO) classification of central nervous system (CNS) tumors introduced the new tumor type CNS tumor with *BCOR* internal tandem duplication (ITD), characterized by a distinct DNA methylation profile and peculiar histopathological features, including a circumscribed growth pattern, ependymoma-like perivascular pseudorosettes, microcystic pattern, absent or focal GFAP immunostaining, OLIG2 positivity, and BCOR immunoreactivity. We describe a rare case of a CNS tumor in a 45-year-old man with histopathological and immunohistochemical features overlapping the CNS tumor with *BCOR* internal tandem duplication (ITD) but lacking BCOR immunostaining and *BCOR* ITD. Instead, the tumor showed *CREBBP::BCORL1* fusion and pathogenic mutations in *BCOR* and *CREBBP*, along with a DNA methylation profile matching the “CNS tumor with EP300:BCOR(L1) fusion” methylation class. Two CNS tumors with fusions between *CREBBP*, or its paralog *EP300*, and *BCORL1*, and approximately twenty CNS tumors with *CREBBP/EP300::BCOR* fusions have been reported to date. They exhibited similar ependymoma-like features or a microcystic pattern, along with focal or absent GFAP immunostaining, and shared the same DNA methylation profile. Given their morphological and epigenetic similarities, circumscribed CNS tumors with *EP300/CREBBP::BCOR(L1*) fusions and CNS tumors with *BCOR* ITD may represent variants of the same tumor type. The ependymoma-like aspect coupled with the lack of diffuse GFAP immunostaining and the presence of OLIG2 positivity are useful clues for recognizing these tumors in histopathological practice. The diagnosis should be confirmed after testing for *BCOR(L1)* gene fusions and *BCOR* ITD.

## Introduction

In recent years, analysis of DNA methylation profiles in central nervous system (CNS) tumors has led to the discovery of new tumor types that exhibit distinct genetic alterations [2]. In 2016, Sturm et al. demonstrated that a portion of tumors, which had been previously diagnosed as primitive neuroectodermal tumors (PNETs), had a distinct DNA methylation profile and were characterized by exon 15 internal tandem (ITD) duplication of *BCOR* [8]. This new molecular entity was designated CNS high-grade neuroepithelial tumor with *BCOR* alteration (CNS HGNET-BCOR) [8]. Subsequent studies have confirmed that these tumors are characterized by peculiar histopathological and immunohistochemical features, including circumscribed growth, ependymoma-like perivascular pseudorosettes, delicate branching capillaries, microcystic pattern, focal or absent GFAP immunostaining, focal OLIG2 positivity, variable expression of NeuN, and immunoreactivity for BCOR [1, 3, 17]. Owing to their unique features, these tumors were considered a novel tumor type named “CNS tumor with *BCOR* ITD” in the fifth edition of the World Health Organization (WHO) classification of CNS tumors [13].

After the first description of CNS tumor with *BCOR* ITD [8], other tumors with overlapping morphological and immunohistochemical features, but harboring fusions of *BCOR* with either *EP300* or its paralog *CREBBP*, instead of *BCOR* ITD, have been reported in the CNS [7, 9, 11, 12, 14, 15]. These tumors exhibit a DNA methylation profile close to that of CNS tumors with *BCOR* ITD, suggesting a similarity between the two [9, 11, 14, 15]. However, *CREBBP/EP300::BCOR* fusions have also been observed in tumors that show morphological features consistent with diffuse gliomas, exhibit extensive immunoreactivity for glial markers, lack immunostaining for NeuN, and have a methylation profile close to low-grade diffuse gliomas [7, 12]. These findings suggest that fusion of *BCOR* with *EP300* or *CREBBP* may contribute to the development of different CNS tumors.

*BCORL1* encodes a protein homolog of BCOR that binds to and interacts with histone deacetylases to repress gene transcription [6]. In a recent study, Yamazaki et al. described a case of diffuse glioma harboring a *CREBBP::BCORL1* fusion, which had not been previously reported in CNS tumors [16].

In this report, we describe the radiological, histopathological, immunohistochemical, and molecular features of another brain tumor that features a *CREBBP::BCORL1* fusion, co-occurring with pathogenic mutations in *BCOR* and *CREBBP*.

## Case presentation

**Fig. 1:**
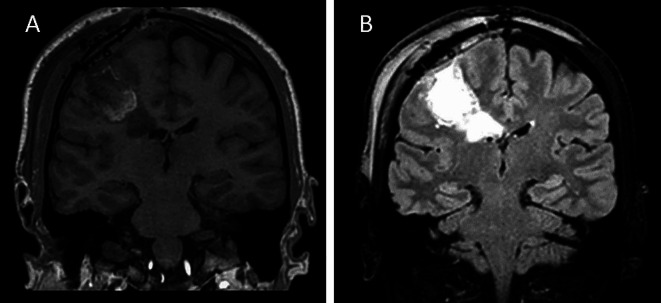
Brain magnetic resonance imagining showing a mass in the frontal lobe, inhomegenously intense in T1 weighted sequences (**A**) and hyperintense in T2 weighted sequences (**B**)

A forty-five year-old man was referred to our hospital due to the onset of a progressive palsy affecting the left upper limb. Brain magnetic resonance imaging (MRI) revealed a demarcated lesion with inhomogeneous contrast enhancement in the right frontal lobe **(**Fig. 1**)**. The lesion measured 42 × 41 × 33 mms and displaced the left lateral ventricle. Under the suspicion of high-grade glioma, the patient underwent surgery with gross total resection of the contrast-enhanced portion of the tumor. The remaining non-enhancing tumor, which was situated in close proximity to the posterior arm of the internal capsule and extended into the body of the right lateral ventricle, was not removed.

Surgical specimens were formalin-fixed and paraffin-embedded for subsequent histopathological evaluation, immunohistochemistry, and cytogenetic and molecular analyses.

**Fig. 2:**
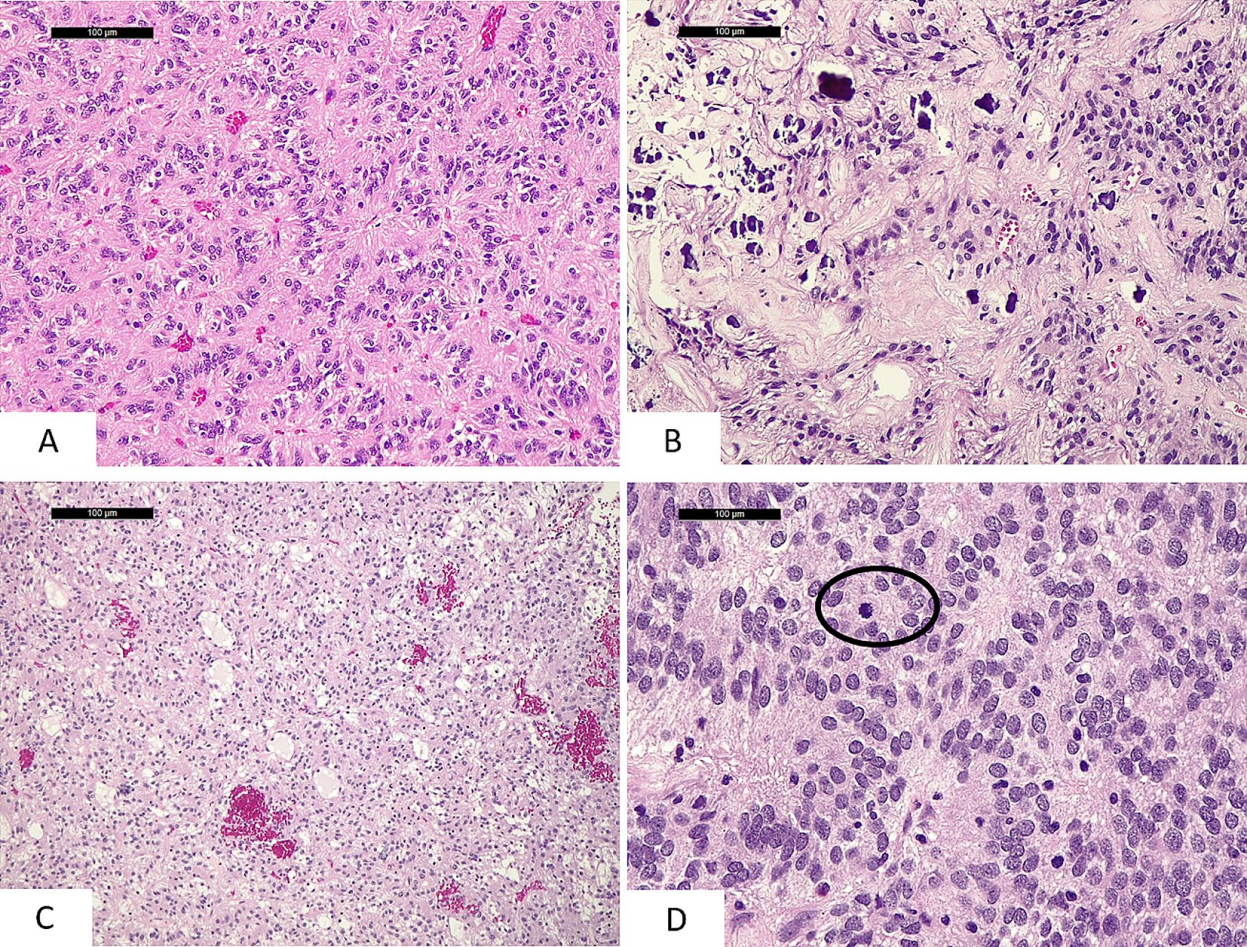
Histopathological features of the tumor consisting in the presence of perivascular pseudorosettes (**A**), calcifications and hyalinized vessels (**B**), microcystic pattern reminiscent of glioma (**C**), areas with increased cellularity and mitoses (**D**) (mitosis within the black circle)

Microscopic examination revealed a neoplasm that featured an expansive growth pattern, had frequent calcifications and hyalinized vessels, and was composed of cells with ovoid nuclei arranged around vascular structures, forming perivascular pseudorosettes. The latter were devoid of the anucleated peri-vascular fibrillary zones typically observed in ependymomas and were formed by tapered neoplastic cell processes anchored to blood vessels **(**Fig. 2**)**. In other areas, the tumor demonstrated a microcystic pattern and features reminiscent of a glioma (Fig. 2), or showed increased cellularity and brisk mitotic activity (Fig. 2**)**. Necrosis was not observed, whereas microvascular proliferation was observed only in adjacent brain parenchyma. Histopathological features suggested ependymoma or a CNS tumor with *BCOR* internal tandem duplication [13].

**Fig. 3:**
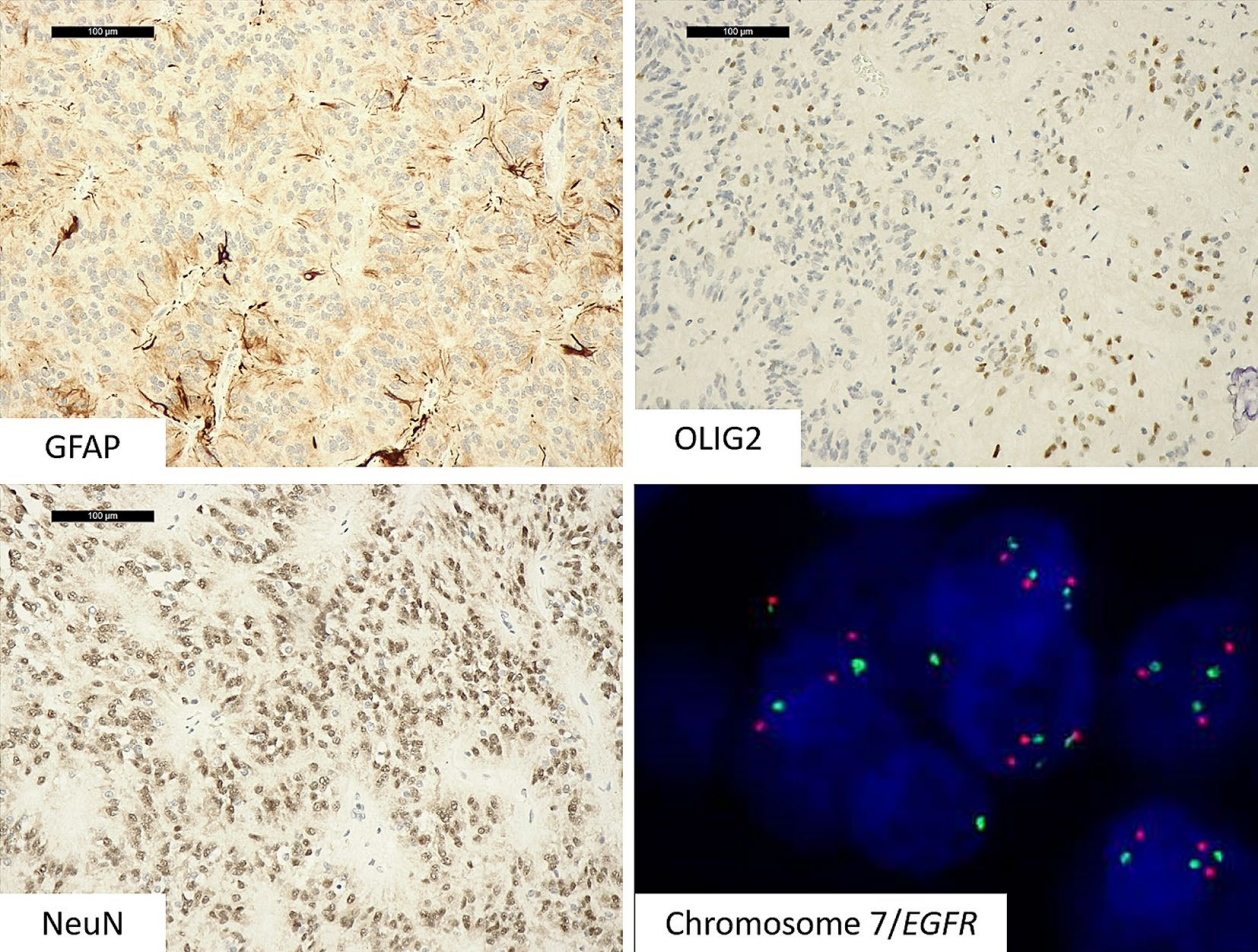
The tumor exhibited only focal immunostaining for GFAP, scattered nuclei positive for OLIG2 and extensive NeuN immunostaining. FISH analysis documented gains of chromosome 7 (red probe: EGFR locus; green probe: chromosome 7 centromere)

Immunohistochemistry revealed that the tumor exhibited focal GFAP immunoreactivity. In contrast to the diffuse positivity typically observed in ependymomas, perivascular pseudorosettes were GFAP-negative. OLIG2 was positive in a subset of tumor cell nuclei. The tumor cells were uniformly positive for NeuN, and negative for EMA, CD34, BCOR, IDH1 p. R132H, and BRAF p. V600E. Neurofilament immunostaining confirmed the lack of infiltrating growth. The Ki-67 labeling index was 10% in areas with the highest cellular density (Fig. 3).

Fluorescence In Situ Hybridization (FISH) failed to demonstrate the rearrangement of *c11orf95* (break-apart probe, Empire Genomics, New York, USA) or *MN1* (break-apart probe, Empire Genomics, New York, USA). It revealed chromosome 7 gains (*EGFR*/CEN 7 dual-color probes, ZytoVision, Bremerhaven, USA) in the absence of chromosome 10 losses (*PTEN*/CEN 10 dual-color probe, ZytoVision, Bremerhaven, USA) (Fig. 3).

**Fig. 4:**
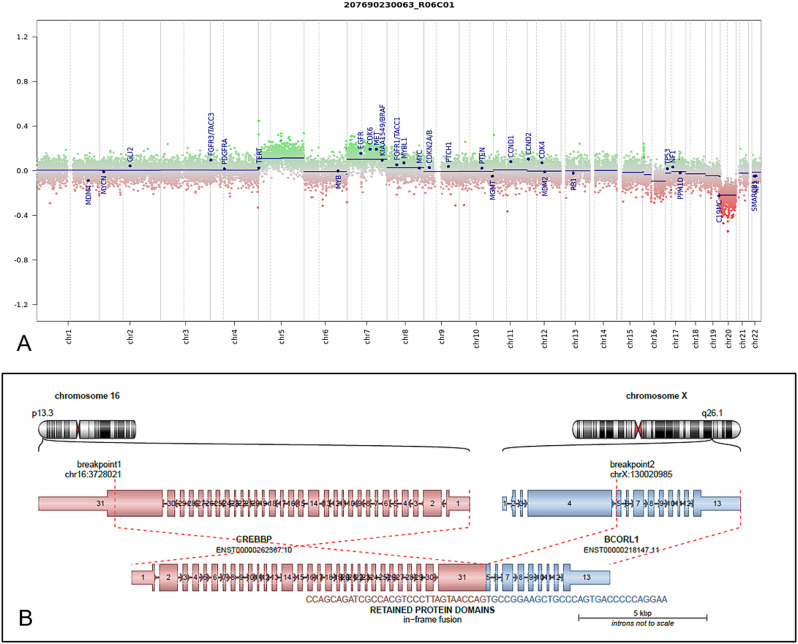
(**A**) Copy number variation plot obtained from DNA methylation analysis showing gains of chromosome 7 and loss of chromosome 20. (**B**) *CREBBP::BCORL1* fusion identified by means of RNA sequencing

Methylation profiling was performed on DNA extracted from formalin-fixed paraffin-embedded tissue sections in enriched tumor areas (tumor purity > 90%) and processed using Infinium Methylation EPIC BeadChip (850k) array (Illumina). By means of the methylation classifier v. 12.5 (available at “hhtps://www.molecularneuropathology.org/”), the tumor was assigned to the methylation family CNS *BCOR*-altered tumors (calibrated score: 0.99), and to the methylation class CNS tumor with *EP300:BCOR(L1)* fusion (calibrated score: 0.90). High-density DNA methylation arrays allowed for the determination of copy number alterations that were consistent with the gain of chromosome 7, loss of chromosome 20, and no other relevant chromosomal aberrations (Fig. 4).

The DNA methylation profile prompted us to investigate the presence of gene fusions, mutations, and copy number variations.

The lesion was first analyzed with an RNA-targeted panel (Custom Archer Fusion Panel) which yielded a negative result. However, whole RNA sequencing (SureSelectXT HS2, Agilent Techonologies) revealed an *in-frame* fusion *CREBBP (exon 31):: BCORL1 (exon 5)* (Fig. 4), which was further confirmed by RT-PCR and Sanger sequencing.

A DNA-targeted panel investigating more than 500 genes (TruSight Oncology 500, Illumina) identified a pathogenic inactivating mutation in *BCOR* (p. P624Rfs*45; variant allele frequency: 71.6%), which was predicted to result in a truncated protein devoid of both the putative nuclear localization signals (i.e., NLS1 and NLS2), possibly accounting for the absence of BCOR nuclear staining observed in this case [10]. Furthermore, an additional pathogenic inactivating mutation was found in *CREBBP* (p. G757Afs*19; variant allele frequency: 24.7%).Tumor Mutational Burden was intermediate (between 6 and 19 mutations/Mb) and MSI stable (3.2%).

One month after surgery, no tumor cells were found on cytological examination of the cerebrospinal fluid, and MRI did not reveal nodular seeding in the cerebral/cerebellar or spinal subarachnoid space. Tumor residue did not show any volumetric increase. The patient was treated with temozolomide and radiotherapy.

## Discussion and conclusions

We report a case of supratentorial brain tumor with *CREBBP::BCORL1* fusion.

In this case, radiological, histopathological, and immunohistochemical features were similar to those previously reported in CNS tumors with *BCOR* ITD, including circumscribed growth, ependymoma-like perivascular pseudorosettes, microcystic areas with a glioma-like appearance, absence of EMA immunostaining, focal positivity for GFAP, and immunoreactivity for OLIG2 and NeuN [13]. Confirming its similarity to CNS tumors with *BCOR* ITD, the tumor was classified into the methylation family of *BCOR*-altered tumors using DNA methylation profiling and v. 12.5 of the classifier. However, consistent with the presence of a fusion involving *BCORL1* and the *EP300* paralog *CREBBP*, the methylation class was “CNS tumor with *EP300*::*BCOR*(L1) fusion”, which was added to the methylation family of BCOR-altered tumors in the v. 12.5.

*CREBBP::BCORL1* fusion is a rare genetic event first identified in 2 cases of ossifying fibromyxoid tumors of soft tissues [5]. To our knowledge, *CREBBP::BCORL1* has been described only in one case of the CNS interpreted as a diffuse glioma [16]. In addition, *BCORL1* fusion with *EP300* has been reported in a CNS tumor originally diagnosed as an anaplastic ependymoma [4]. Notably, both cases aligned with the methylation class “CNS tumor with *EP300*::*BCOR*(L1) fusion [4, 16], similar to the present case. The original diagnosis of anaplastic ependymoma in the case with *EP300::BCORL1* fusion, which was characterized by perivascular pseudorosettes and calcifications, highlights its morphological similarity to the present case [4]. Although the tumor with *CREBBP::BCORL1* fusion was described as a diffuse glioma owing to an infiltrative growth pattern, it also had several histopathological and immunohistochemical similarities with the present case, including a microcystic pattern, presence of calcifications, and only focal GFAP staining, along with the immunoreactivity for OLIG2 and a neural marker [16]. Gain of chromosome 7 was present in both previous CNS tumors with *BCORL1* fusion [4, 16], as in the present case, suggesting that this could represent a distinctive feature of these tumors (See Table 1).

**Table 1:** Clinical, immunohistochemical and molecular data of the three CNS tumors with *CREBBP(EP300)*: *BCORL1* fusion, including the present case

Sex /age	Site	Original diagnosis	IHC	BCOR nuclear expression	BCORL1 fusion	DNA Methylation class	CNV	Follow-up (mo)	Reference
M/45	Fr	CNS tumor *CREBBP::BCORL1*	GFAP-/+, OLIG2-/+, NeuN+/- EMA -	No	*CREBBP (ex 31):: BCORL1(ex5)*	CNS tumor with *EP300::BCOR(L1)* fusion (0.90, v12.5)	Chr 7 gain, chr 20 loss	Recent case	Current study
F/17	Fr	Diffuse glioma	GFAP-/+, OLIG2+, EMA -	Yes	*CREBBP (ex 31):: BCORL1(ex6)*	CNS tumor with *EP300::BCOR(L1*) fusion (0.99, v12.5)	Chr 7 gain	Multiple R (18,33,89) DOD (115)	[16]
M/72	O	Ependymoma grade 3	NA	NA	*EP300 (ex 31):: BCORL1(ex4)*	CNS tumor with *EP300::BCOR(L1)* fusion (0.98, v12.5)	Chr 7 gain, gain at chr 1q, 3p, 9q	R (24), alive (33)	[4]

Fr: frontal lobe; O: occipital lobe; NA: not available; R: relapse; Mo: months; CNS HGNTBCOR: CNS high grade neuroepithelial tumor with BCOR alteration (v11b4 classifier); DOD: Dead of disease

CNS tumors harboring fusions of *CREBBP* or *EP300* with *BCOR*, instead of *BCORL1*, seem to be slightly more frequent, with twenty-three cases reported thus far [7, 9, 11, 12, 14, 15]. Nineteen of these tumors displayed a DNA methylation profile matching the methylation class “CNS tumor with *EP300:BCOR(L1)* fusion” [9, 11, 14, 15] and had radiological, histopathological, and immunohistochemical features overlapping with those reported in this and in other cases with *BCORL1* fusion or in CNS tumors with *BCOR* ITD. They mainly featured a circumscribed growth pattern, frequent ependymoma-like morphology, focal-to-absent immunostaining for GFAP, and positivity for OLIG2 and neural markers [9, 11, 14, 15]. However, in contrast to CNS tumors with *BCOR* ITD, which are invariably BCOR-positive [13], CNS tumors with *BCOR* fusions were either immunohistochemically positive or negative for BCOR [14]. Similarly, while a diffuse nuclear BCOR expression was described in the previously reported case harboring *CREBBP::BCORL1* fusion [16], the current case did not show any nuclear positivity, thus qualifying the tumor as negative for BCOR staining. This finding may be possibly related to the co-occurring truncating *BCOR* mutation because the predicted protein does not contain the putative nuclear localization signals [10].

Despite their morphological similarities, CNS tumors with *BCOR(L1)* fusions and those with *BCOR* ITD appear to have distinct clinical features. The former seem to affect older patients than the latter; indeed, the median age at onset was 30 years (range 5–72 years) in the 19 tumors with *BCOR* fusions [9, 11, 14, 15] versus 4 years (range 2–44 years) in the 15 patients with CNS tumors harboring *BCOR* ITD [14]. Interestingly, the 3 cases of CNS tumor with *BCORL1* fusion described so far affected patients of any age, i.e. a 17-year old [16], a 45 year-old (present case) and a 72-year old [4] patient. CNS tumors with *BCOR(L1*) fusions are localized in the cerebral lobes or posterior fossa, similar to CNS tumors with BCOR ITD [9, 11, 14, 15]. However, the latter may also rarely occur in the brainstem or spinal cord [8].

The limited available data suggests that CNS tumors with *BCOR* fusion have a slightly better prognosis than CNS tumors with *BCOR* ITD. Indeed, in 17 patients with a CNS tumor harboring *CREBBP*/*EP300::BCOR* fusion and a DNA methylation profile of “CNS tumor with *EP300:BCOR(L1)* fusion”, the median progression-free survival was 16 months, and the recurrence rate was 35% (6/17) [9, 11, 14, 15]. In contrast, in 26 CNS tumors with *BCOR* ITD the median progression-free survival was 12.5 months, and the recurrence rate was 65% (17/26) [11].

Concerning CNS tumors with *BCORL1* fusion, while our case is too recent to report outcome data, both the previously described patients developed tumor recurrence after an interval of 23 [4] and 18 months [17], with one patient alive 33 months after diagnosis [4], and the other dying at 115 months [16], respectively (Table 1).

Notably, *EP300/CREBBP::BCOR* fusions have also been reported in four CNS tumors showing a DNA methylation profile that does not match any methylation class, but is in close proximity to gliomas with *MYB/MYBL1* alterations [12, 14]. These tumors were diagnosed in children or adolescents and displayed radiological and histopathological features consistent with diffuse gliomas, widespread immunostaining for GFAP, OLIG2, and BCOR, and immunohistochemical negativity for NeuN and other neuronal markers [12]. Mutation in *pTERT* along with *IDH* wild-type status in one of these cases would be compatible with the diagnosis of diffuse astrocytoma with molecular features of glioblastoma IDH-wildtype according to the current WHO criteria [7]. In another case, the histopathological features suggested a high-grade glioma, whereas the remaining two cases were histologically most consistent with glioneuronal tumors [12]. Owing to the limited number of described cases and limited follow-up time, it is unclear whether these tumors have a different prognosis from the other CNS tumors with *BCOR(L1)* fusions that fall into the methylation class “CNS tumor with *EP300:BCOR(L1*) fusion”.

In conclusion, we described the second case of a CNS tumor with *CREBBP::BCORL1* fusion. The morphological overlapping features and the similar DNA methylation profile suggest that circumscribed CNS tumors with *BCOR or BCORL1* fusions and CNS tumors with *BCOR* ITD may be genetic variants of the same tumor type. However, based on the limited data, the latter seem to affect younger individuals and to have higher recurrence rate and shorter progression-free survival. The differential diagnosis of CNS tumors with *BCOR(L1)* fusion towards ependymoma may be difficult owing to similar morphological features and the possible absence of BCOR immunostaining. In tumors with ependymoma-like appearance, the focal-to-absent GFAP staining, along with OLIG2 and NeuN positivity, and absence of *ZFTA* or *YAP1* fusions, suggest a BCOR-altered tumor and should prompt the investigation *BCOR(L1)* fusions and, eventually, the analysis of DNA methylation profiling.

## Data Availability

Data are available from the corresponding author on reasonable request.
